# Nasal irrigation with licorice extract (*Glycyrrhiza glabra*) in treating nasal polyps by reducing fibroblast differentiation and extracellular matrix production in TGF-β1-stimulated nasal polyp-derived fibroblasts by inhibiting the MAPK/ERK-1/2 pathway – an in vitro and *in clinic* study

**DOI:** 10.1186/s12906-022-03791-y

**Published:** 2022-11-29

**Authors:** Geng-He Chang, Pei-Rung Yang, Yu-Ching Cheng, Ke-Hsin Hsu, Ching-Yuan Wu, Yao-Hsu Yang, Yu-Shih Lin, Cheng-Ming Hsu, Ming-Shao Tsai, Yao-Te Tsai, Pey-Jium Chang

**Affiliations:** 1 Department of Otolaryngology, Chang Gung Memorial Hospital, Chiayi, Taiwan; 2 Graduate Institute of Clinical Medical Sciences, College of Medicine, Chang Gung University, Taoyuan, Taiwan; 3 Faculty of Medicine, College of Medicine, Chang Gung University, Taoyuan, Taiwan; 4 Head and Neck Infection Treatment Center, Chang Gung memorial Hospital, Chiayi, Taiwan; 5 Department of Traditional Chinese Medicine, Chang Gung Memorial Hospital, Chiayi, Taiwan; 6 School of Traditional Chinese Medicine, College of Medicine, Chang Gung University, Taoyuan, Taiwan; 7 Department of Pharmacy, Chiayi Chang Gung Memorial Hospital, Chiayi, Taiwan

**Keywords:** Licorice, *Glycyrrhiza glabra*, Nasal irrigation, Nasal polyp, Sinusitis, Fibroblast

## Abstract

**Background:**

To date, treating nasal polyps (NPs) is still a medical challenge. However, we have developed an innovative therapy using licorice extract (LE: *Glycyrrhiza glabra*) to treat rhinitis and sinusitis via nasal irrigation and have discovered that it significantly affects treatment of NPs.

**Hypothesis/purpose:**

This study investigated the mechanism of LE on NPs.

**Study design:**

NPs were collected from three patients using tissue biopsies before and 2 weeks after nasal irrigation with licorice for histopathological analysis. Additionally, NPs from two patients were collected, and nasal polyp-derived fibroblasts (NPDF) were isolated and cultured.

**Methods:**

The TGF-β1-stimulated NPDF model was used to examine the effect of LE on fibroblast differentiation (biomarker: α-SMA), the consequent production of extracellular matrix (ECM; biomarkers: fibronectin, FBN), and the functional signaling pathway.

**Results:**

Immunohistochemistry (IHC) revealed that the number of eosinophils and the expression of α-SMA and interstitial collagen of polyps after licorice treatment significantly decreased. Additionally, RT-PCR, western blotting, and immunofluorescence (IF) showed that α-SMA and FBN expressions were significantly increased in the NPDF, which was stimulated by TGF-β1, and LE dose-dependently could effectively reduce this effect. Furthermore, western blotting showed that LE could attenuate α-SMA and FBN expressions by preventing the signaling pathway of MAPK/ERK-1/2, which IHC and IF further confirmed. In addition, LE effectively suppressed the cell migration of NPDF, which is related to polyp expansion.

**Conclusion:**

LE is clinically used to treat sinusitis with NPs through nasal irrigation, which significantly reduces the size of NPs. This effect could attenuate fibroblast differentiation, ECM production and cell migration, and one of the functional mechanisms may be through inhibition of the MAPK/ERK-1/2 signaling pathway.

**Trial registration:**

ISRCTN (No. 51425529) registered on 17/04/2020 (retrospectively registered) - http://www.isrctn.com/ISRCTN51425529

**Supplementary Information:**

The online version contains supplementary material available at 10.1186/s12906-022-03791-y.

## Introduction

Nasal polyps (NPs) are a chronic inflammatory disease of the nasal cavity and sinuses that affect the mucosal remodeling process and produces non-neoplastic mucosal changes, resulting in NPs. Additionally, NPs can block the opening of the sinuses and sometimes the sinuses themselves, which can result in the blockage of the circulatory secretion of the sinuses and consequent chronic sinusitis [1]. Moreover, NPs are highly prevalent in many countries of the world, affecting vast groups of patients and seriously affecting their quality of life. Therefore, the lack of effective treatment may lead to lower airway complications, such as asthma [2].

According to the recent treatment guidelines issued by the American Academy of Otorhinolaryngology, topical steroid sprays and saline nasal irrigation are recommended for treating chronic rhinosinusitis (CRS) with NPs (CRSwNP) or without NPs [3]. However, medical therapeutic efficacy is often limited to treating patients with extensive NPs [4] and patients with refractory CRSwNP who, when medical management fails, are often evaluated for endoscopic sinus surgery (ESS) [5]. Although ESS is a highly successful technique to improve the subjective symptoms and objective findings of the endoscopic examination or computed tomography scan, high postoperative recurrence of NPs, which often leads to refractory sinusitis, remains a challenging issue for otolaryngologists [6]. Occasionally, revised surgery and administration of oral steroids are recommended to control the symptoms, although these treatments can adversely affect patients’ psychology and physiology [7]. Several methods have been proposed to improve the therapeutic efficacy of controlling NPs, including immunotherapy such as anti–immunoglobulin E (omalizumab) [8], anti–interleukin–5 (mepolizumab) [9], and anti–IL4 receptor (dupilumab) [10] humoral antibodies. However, managing NPs remains difficult to date. Therefore, a significant breakthrough in treating NPs is an important goal.

**Fig. 1:**
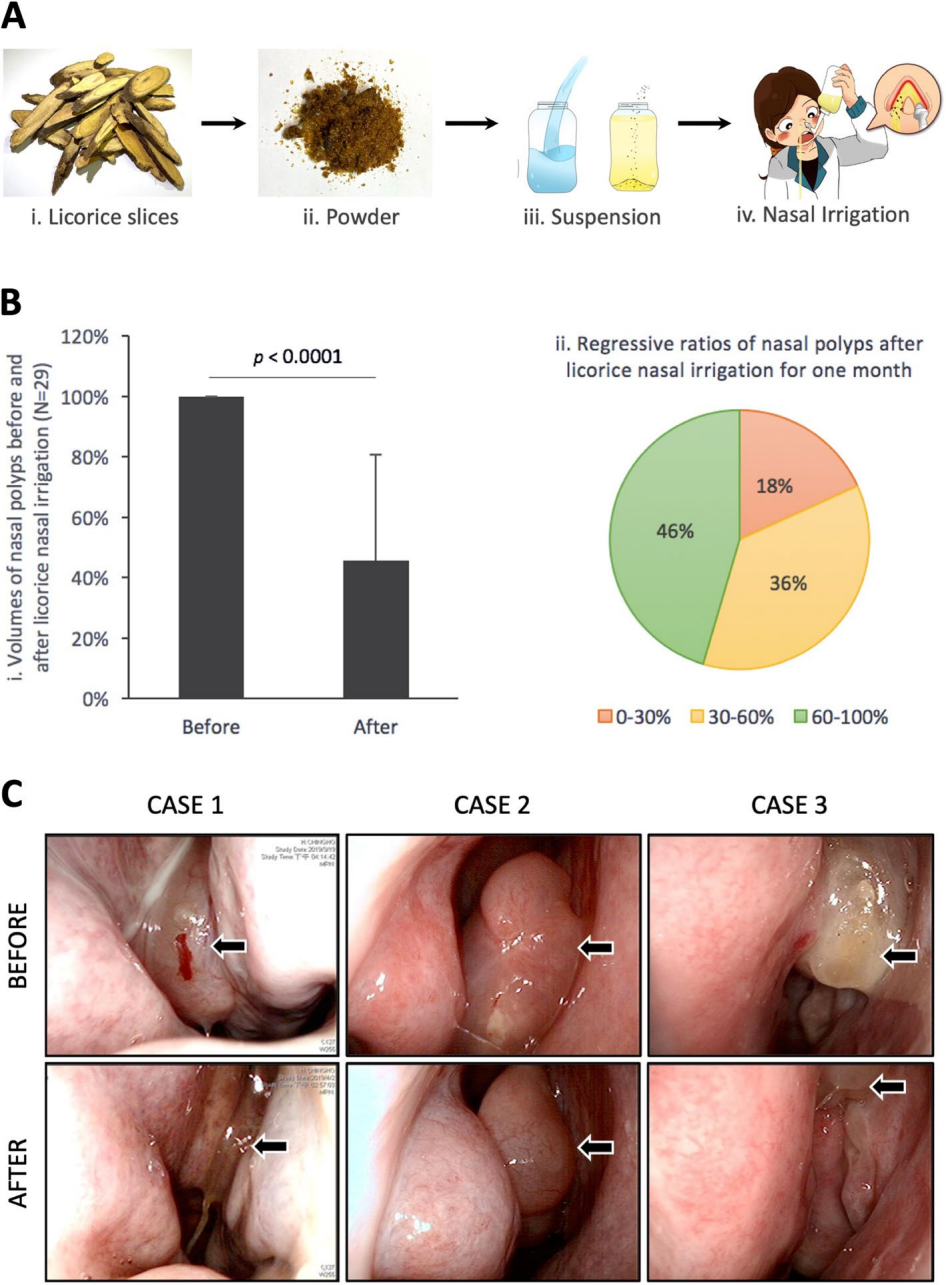
**A** Preparation and operating processes of nasal irrigation with licorice extract. (i) Sections of licorice root (*Glycyrrhiza glabra*) boiled in a high-pressure cooker, then filtered, and concentrated to obtain the crude extract; (ii) the crude extract processing lyophilization to obtain licorice powder; (iii) the licorice powder was added to a squeeze bottle and mixed with warm water to form a licorice suspension with 3 mg/mL concentration; (iv) the prepared licorice extract used to clean the nasal passages and treat sinonasal diseases through nasal irrigation. **B** Therapeutic effect of nasal irrigation with licorice extract on nasal polyps. (i) Overall, 29 patients with nasal polyps received 1 month of nasal irrigation with licorice extract. The cross-sectional area of their polyps was recorded and measured using video-endoscopy. The changes in the area before and after treatment were also compared. (ii) The degree of area reduction of the nasal polyp images after treatment. They were categorized into three groups (0–30%, 30–60%, and 60–100%), and the proportions of the three groups were calculated. **C** Endoscopic findings in patients with nasal polyps under nasal irrigation with licorice extract. Three patients with nasal polyps underwent nasal irrigation with licorice extract for one month, and video-endoscopy was performed to image-record the changes in size of their nasal polyps (black arrows) before and one month after treatment

Nasal irrigation using isotonic saline solution has been shown as an effective adjunctive treatment for CRS [11]. However, because its therapeutic benefits and length of controlling rhinitis and sinusitis-associated symptoms are limited, many previous studies have found that adding anti-inflammatory agents such as corticosteroids to nasal irrigation can improve CRS treatment [12]. Recently, we used cell and animal experiments to develop a designed licorice (*Glycyrrhiza glabra*) extract (LE) to treat rhinitis and CRS through nasal irrigation (Fig. 1A). The results from this clinical trial showed that this method is significantly better than saline nasal irrigation for the relief of rhinitis-associated symptoms and better than steroid nasal irrigation in the subjective and objective evaluation. Additionally, this innovative method can significantly decrease patients’ nasal discomfort during nasal irrigation [13].

In our clinical trial, 29 patients with CRSwNP completed 1 month of nightly nasal irrigation with licorice (LNI). After analyzing the difference in the size of NPs before and after treatment based on the endoscopic images [13], the results revealed that the area decreased by 60–100% (significant regression) in 46% of those patients with CRSwNP, decreased by 30–60% (moderate regression) in 36%, and decreased by 0–30% (slight regression) in 18%. Overall, the polyps in 29 patients had a 55% reduction in cross-sectional area under endoscopy (*p* < 0.0001) (Fig. 1B). Therefore, the novel method of nasal irrigation can contribute a highly significant effect in the treatment of NPs in the clinic. Therefore, through this study, we examined the functional mechanism based on the clinical effect of the LE in treating NPs.

## Materials and methods

The development of NPs is generally caused by stimulation on the epithelial mucosa of nasal cavity by inflammatory substances that destroy the epithelium and subsequently produce subepithelial mesenchyme to generate a remodeling process, which results in the deformation and protrusions to form a spherical object that causes nasal congestion and the obstruction of the sinus opening [1].

In cell-level studies, the generally used model for research includes isolated fibroblasts from NPs (NP-derived fibroblast, NPDF) [14, 15]. However, under transforming growth factor–β1 (TGF-β1) stimulation [16, 17], the downstream inflammatory pathways are activated, which include mitogen-activated protein kinase/extracellular signal-regulated kinases (MAPK/ERK), p38, and c-Jun N-terminal kinase (JNK), among others [14, 18, 19], producing α-smooth muscle actin (α-SMA) to convert fibroblasts into myofibroblasts [20]. Subsequently, the activated fibroblasts generate extracellular matrix (ECM), containing fibronectin (FBN) and type I collagen, among others, which could increase the mesenchyme of polyps [19]. Therefore, this study was based on the commonly used NP-experimental model for analysis.

**Fig. 2:**
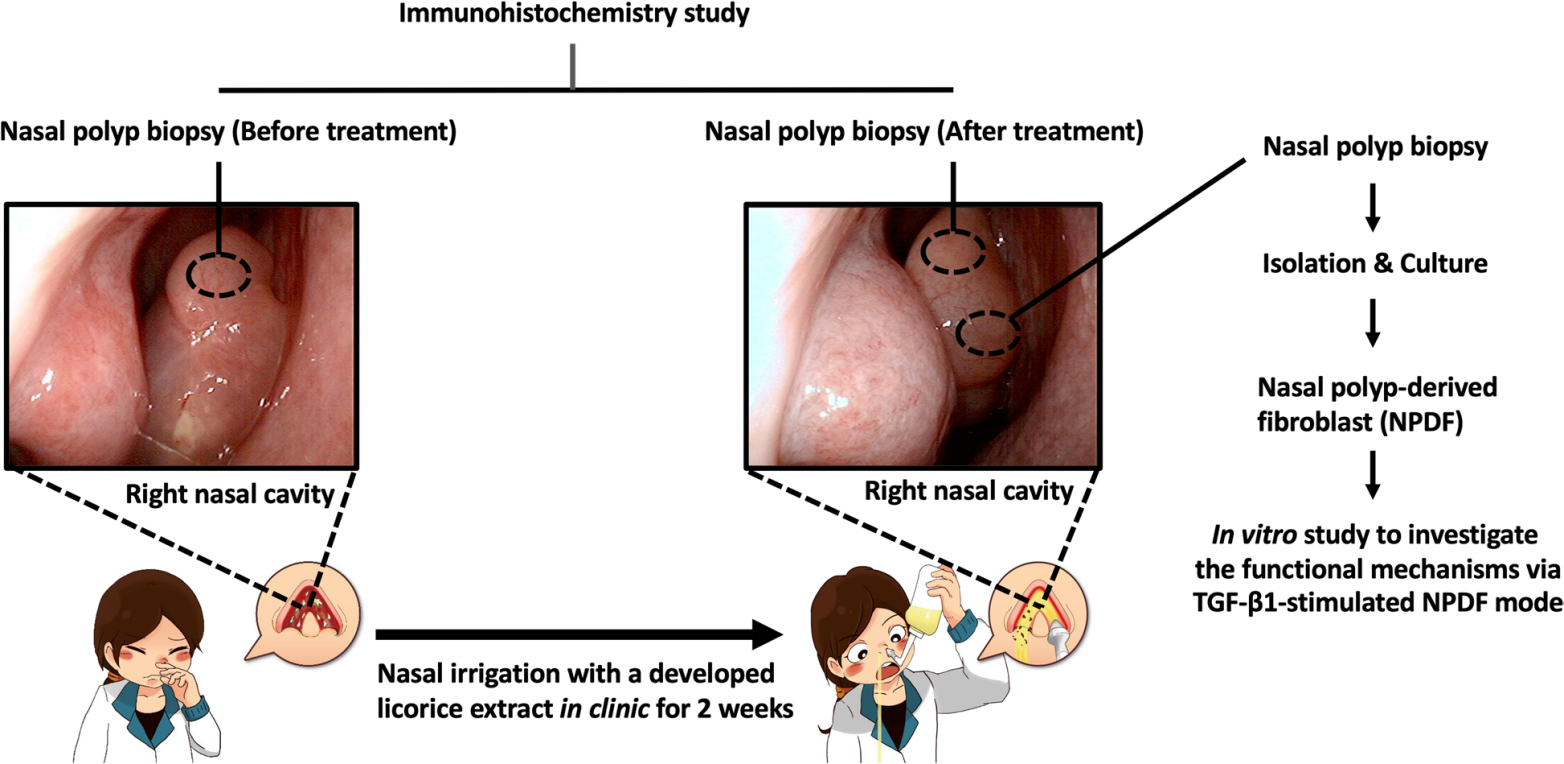
Research process. Patients with nasal polyps received biopsies before and two weeks after using nasal irrigation with licorice extract, and the fibroblasts in the polyps were isolated for culture (*n* = 3). The pathological differences of nasal polyps before and after treatment were evaluated by immunohistochemistry staining, and the molecular mechanism of the licorice extract on fibroblasts was investigated by in vitro experiments

We enrolled three patients with large NPs extending to the nasal cavities who received LNI treatment and endoscopic examination before and after treatment showed that the sizes of NPs had regressed significantly (Fig. 1C). Biopsies under endoscopy were performed for the NPs 2 weeks before and after the licorice treatment (Fig. 2).

### H&E histology

Specimens of NPs before and after treatment with LE were stained with hematoxylin and eosin (H&E), and the numbers of eosinophils, lymphocytes, neutrophils and plasma cells were calculated and analyzed as follows: in each patient’s NP specimen, under 400× microscopic view, we selected three separate full-fields and estimated the numbers of above-mentioned four types of cells under H&E stain, respectively, and subsequently obtained a total of nine results from the three patients. The results obtained from the nine visual fields were averaged, and the differences in the numbers of eosinophils, lymphocytes, neutrophils and plasma cells before and after treatment were analyzed.

### Immunohistochemistry

The differences of TGF-β1 (BS1361; Bioworld Technology, Inc., MN, USA), α-SMA (Sigma-Aldrich Corp., St. Louis, MO, USA), vimentin (Cell Signaling), Masson’s trichrome (TASS01; Toson Technology Co., Ltd., Hsinchu, Taiwan), ERK (Cell Signaling), and phosphorylated ERK (p-ERK) (Santa Cruz Biotechnology, TX, USA) staining in those NP specimens before and after licorice treatment were analyzed by immunohistochemistry (IHC).

Furthermore, the method for analyzing TGF-β1, α-SMA, vimentin, Masson’s trichrome, ERK, and p-ERK included selecting three separate full-fields for each patient’s NP specimen under 400× microscopic view and the subsequent use of ImageJ software to filter the stained area. Next, we calculated the ratio of stained area relative to the entire field of view and averaged the nine data of these three patients. Lastly, the differences between the staining biomarkers before and after treatment were analyzed.

### Nasal polyp-derived fibroblast (NPDF)

Briefly, we collected the NPs from two patients with clinically significant responses to LNI (the cross-sectional area of the polyps in endoscopic images was reduced by 60–100% after treatment) (Fig. 2). After their NPs were separated, the fibroblasts were isolated and cultured to enable subsequent cell experiments. The NPs were obtained during sinonasal surgery and were subsequently processed into single-cell suspensions using a gentle MACS dissociator (Miltenyi Biotec GmbH, Bergisch Gladbach, Germany), which is a specialized machine for processing human nasal mucosa into single-cell suspensions. Furthermore, we sorted the nasal polyp-derived fibroblasts (NPDFs) from the single-cell suspensions. On the basis of the protocol of the EasySep Human-Positive Selection Kit II (STEMCELL Technologies Inc., Vancouver, Canada), the samples were placed into a magnet and then incubated for the sorting process. Next, we sorted and purified the NPDFs using flow cytometry (BD FACS Canto II, BD Biosciences, San Jose, CA, USA) with fluorochrome-conjugated antibody clones, including the anti-human fibroblast antibody (anti-vimentin antibody for human, Abcam, Cambridge, MA, USA) and pan-cytokeratin antibody (Santa Cruz Biotechnology), which was considered the control. After sorting, we further employed flow cytometry to confirm the isolated cells with anti-vimentin antibodies.

### Licorice extract

We prepared the LE for research following our developed standardized protocol [13]. Briefly, 450 g of licorice slices (*Glycyrrhiza glabra*) was put into a 2-L high-pressure cooker, and 1.1 L of reverse osmosis pure water was added. The decoction time was 30 minutes, and the temperature was set to 120 °C. Next, the decoction liquid was carefully filtered to remove impurities, after which we removed water from the liquid using concentration and freeze–drying processes. Approximately 90 g of the dry extract could be obtained (extraction rate of ~ 20%). Finally, we used the dry extract for cell experiments. We used *Glycyrrhiza glabra* for all methods of this study in accordance with the relevant institutional and national, and international guidelines, regulations and legislation.

### Real-time quantitative polymerase chain reaction (RT-PCR)

Briefly, 1 × 10^6^ NPDF cells were seeded into a 10 cm dish and treated with phosphate-buffered saline (PBS) or different concentrations of TGF-β1 (20 ng/ml) and LE (200, 500, 1000 μg/mL) for 24 hours. The experimental groups included the following: 1. PBS as a control; 2. TGF-β1 alone; 3. LE alone; and 4. TGF-β1 and LE. On the basis of the manufacturer’s instructions, we applied the RNA isolation protocol to extract the RNA (NucleoSpin RNA Midi kit, MACHEREY-NAGEL GmbH & Co. KG, Duren, Germany). Subsequently, the first-strand cDNA fragments were synthesized using cDNA synthase (iScript™ cDNA Synthesis Kit, Bio-Rad Laboratories, Inc., Hercules, CA, USA) in a cDNA synthesizer (C1000 Thermal Cycler, Bio-Rad Laboratories). Next, the cDNA was transferred to the RT-PCR workstation (CFX96 Real-Time System, Bio-Rad) to synthesize the target DNA using various gene-specific primers, which include the following: α-SMA (sense sequence 5′–TGA AGT ACC CGA TAG AAC ATG–3′ and antisense sequence 5′–ATG CCA GTT GTG CGT CCA GAG–3′), FBN (sense sequence 5′–TGG TTG TAT CAG GAC TTA TGG–3′ and antisense sequence 5′–CCT GTG ATG GTG TAG CTT CTG–3′), collagen type I (sense sequence 5′–TGG TTG TAT CAG GAC TTA TGG–3′ and antisense sequence 5′–CCT GTG ATG GTG TAG CTT CTG–3′), and glyceraldehyde-3-phosphate dehydrogenase (GAPDH; sense sequence 5′–AGA ACA TCA TCC CTG CCT CT–3′ and antisense sequence 5′–TTA CTC CTT GGA GGC CAT GT–3′). Additionally, the values of the threshold cycle (Ct) were identified in the individual reactions using the CFX96 Real-Time System software. Finally, the Ct values of GAPDH were used as controls and normalization, and relative mRNA expressions were calculated using the 2^−ΔΔCt^ method.

### Western blotting

Briefly, 6 × 10^5^ NPDF cells were seeded in a 6 cm dish and treated or untreated with TGF-β1 (20 ng/mL) for 1 hour. Subsequently, the cells were treated with PBS or different concentrations of LE (25, 100, 300, 1000 μg/mL) in a culture medium for 24 hours and lysed for immunoblotting. The experimental groups included the following: 1. PBS as a control; 2. TGF-β1 alone; 3. LE alone; and 4. TGF-β1 and LE. Next, proteins in the lysate were quantified. Then, aliquots of cellular protein were subjected to SDS-PAGE electrophoresis for western blotting and later transferred to polyvinylidene difluoride membranes (PVDF; Immobilon-P PVDF Membrane, Merck, Darmstadt, Germany). The membranes were probed with the primary antibodies for overnight incubation at 4 °C. The primary antibodies used included the following: α-SMA (Cell Signaling), FBN (Cell Signaling), ERK-1/2 (Cell Signaling), phosphorylated ERK-1/2 (p-ERK-1/2) (Cell Signaling), JNK (Cell Signaling), phosphorylated JNK (p-JNK) (Cell Signaling), p38 (Cell Signaling), phosphorylated p38 (p-p38) (Cell Signaling), and GAPDH (Proteintech, Rosemont, IL, USA). Next, a secondary antibody was added on the basis of the type of primary antibody, and subsequently, a chemiluminescent horseradish peroxidase (HRP) substrate (Themo Fisher or Merck) was added to generate luminescence. Finally, ImageJ software was used for quantification and analysis based on the protein bands and GAPDH as an internal control.

### Immunofluorescence

Additionally, we used immunofluorescence (IF) staining and confocal laser microscopy to detect and analyze the expressions of α-SMA and ECM of the NPDFs stimulated by TGF-β1 (20 ng/ml) and treated with or without LE (500 μg/mL). Briefly, 1.5 × 10^5^ NPDFs were seeded in a six-well plate containing a coverslip per well. Subsequently, PBS or TGF-β1 (20 ng/mL) was added, and the cells were treated with PBS or 500 μg/mL LE for 24 hours. Next, the NPDFs were fixed with 4% paraformaldehyde and added to 0.2% Triton X-100 to increase permeability, followed by the addition of antibodies of α-SMA (Cell Signaling), FBN (Cell Signaling), ERK (Cell Signaling), and p-ERK-1/2 (Cell Signaling), and incubated overnight at 4 °C. Subsequently, rabbit fluorescent antibodies with exciting / absorbing wavelengths of 488 / 500–560 nm were added to these cells for incubation. Finally, the stained NPDFs were visualized and photo-captured using laser scanning confocal microscopy (TCS SP5 II; Leica, Wetzlar, Germany).

### Cell migration

Because fibroblast mobility is associated with polyp volume expansion, we used the transwell migration assay to assess the individual cell migration status of TGF-β1-stimulated NPDF in the presence or absence of LE. 24-well hanging inserts (Millicel Cell Culture Inserts; Merck) with 8 μm mini-holes were used and placed in a 24-well plate. Next, the Roswell Park Memorial Institute (RPMI)-free culture medium was added to the transwell high chamber, and 2 × 10^4^ NPDFs were seeded. The RPMI culture medium was then added to the transwell low chamber. The fibroblasts migrating to the low chamber were fixed with 4% paraformaldehyde after adding PBS or TGF-β1 (20 ng/mL) with PBS or LE (500 μg/mL) for 24 and 48 hours in the upper chamber. Finally, the insert was inverted after adding crystal violet dye (Sigma) to the cells. A light microscope was used to visualize and photo-capture the stained NPDFs from five selected viewpoints.

### Statistical analysis

During IHC, three results were measured for each patient, and a total of nine results were obtained for the three patients. Subsequently, paired Student’s *t*-tests were used for the statistical analysis before and after treatment. In the experimental part of RT-PCR, western blotting, IF, and migration assay, each result was performed at least three times and presented as a mean with standard deviation. Next, the differences between the two groups were evaluated using the unpaired Student’s *t*-test for statistical analysis. Statistically, a significant difference was considered at a *p*-value less than 0.05 and was marked with * on the graph, less than 0.01 as **, and less than 0.001 as ***. Additionally, another group was marked with #, ##, and ### to indicate when two kinds of groups were analyzed together.

## Results

Clinically, we used the developed LE to treat patients with CRSwNP through nasal irrigation and observed that this treatment significantly affects NPs. Therefore, this study used various modalities to assess the microscopic changes and functional mechanisms of LE on NPs.

### Pathologic changes in nasal polyps after nasal irrigation with licorice

**Fig. 3:**
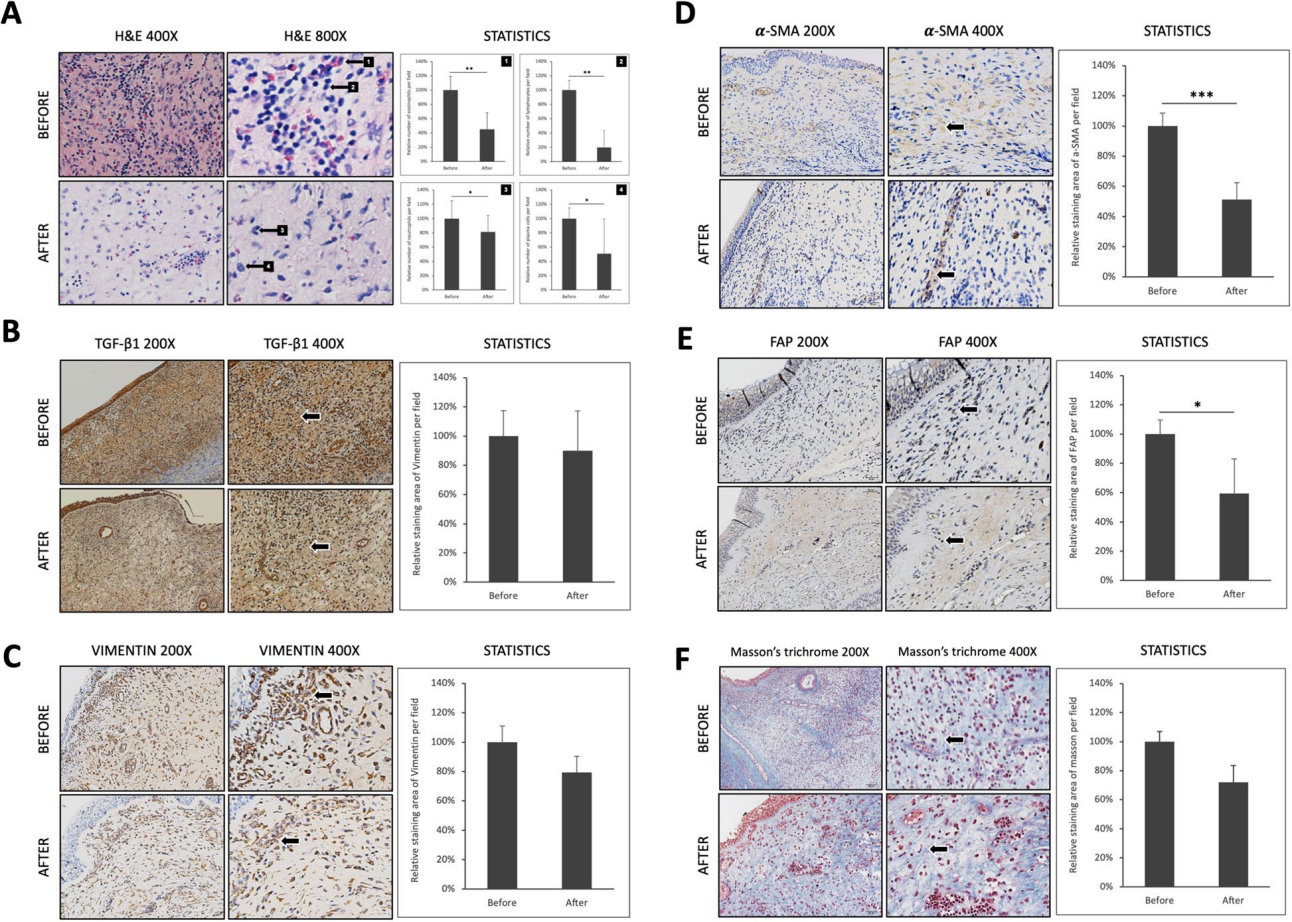
Comparison in histopathology of nasal polyps before and 2 weeks after licorice treatment (*n* = 3). **A** Under H&E staining (400× and 800×), the nasal polyps before licorice treatment were infiltrated with numerous eosinophils (black arrow No.1), lymphocytes (black arrow No.2), neutrophils (black arrow No.3) and plasma cells (black arrow No.4). After licorice treatment for 2 weeks, the relative numbers per field (400×) of eosinophils, lymphocytes, neutrophils and plasma cells reduced by an average of 54.9% (*p* < 0.01), 80.2% (*p* < 0.01), 18.8% (*p* < 0.05) and 49.2% (*p* < 0.05), respectively. **B** Changes in TGF-β1 staining (200x and 400x; black arrows) in nasal polyps before and after treatment were not statistically different. **C** Changes of vimentin staining in specimens before and after treatment. The staining area (black arrows) was relatively reduced by 20% after the treatment, but the difference was not statistically significant. **D** The staining difference in α-SMA in polyp specimens before and after treatment. The statistics showed an average 50% reduction in the stained area (*p* < 0.001). **E** Comparing the staining fibroblast activation protein (FAP) in the nasal polyps before and after treatment. The difference showed a significant 40% reduction of staining area (*p* < 0.05). **F** Differences in Masson’s trichrome staining in the polyp tissue before and after treatment. The results showed an average of 30% reduced stained collagen after treatment (*p* < 0.01)

Various stains were used for the analysis to understand the histopathological changes in clinically significantly reduced NPs. Two weeks after receiving the LNI, the relative numbers per field (400×) of eosinophils, lymphocytes, neutrophils and plasma cells in NPs reduced by an average of 54.9% (*p* < 0.01), 80.2% (*p* < 0.01), 18.8% (*p* < 0.05) and 49.2% (*p* < 0.05), respectively (Fig. 3A). Although there was no significant difference in the staining status of vimentin and TGF-β1 before and after treatment (Fig. 3B–C), α-SMA was significantly reduced by approximately 50% after treatment (*p* < 0.001) (Fig. 3D). Additionally, the staining for fibroblast activation protein (FAP) was significantly reduced by 40% after treatment (*p* < 0.01) (Fig. 3E). The Masson’s trichrome staining for collagen in NP tissue was also significantly reduced by 30% (*p* < 0.01) (Fig. 3F).

The histopathological results indicated that the activating fibroblast biomarker α-SMA, which converted fibroblasts into myofibroblasts for ECM production, and the FAP were both reduced under the licorice treatment. By contrast, the results showed that the stimulating source (TGF-β1) and the fibroblasts stained by vimentin had no significant change. Furthermore, the stroma of NPs (collagen regions stained by Masson’s trichrome) produced by the myofibroblasts decreased. The histopathological results confirmed the macroscopic changes in the volume reduction of NPs observed under endoscopy.

### Effects of TGF-β1 on NPDF for fibroblast differentiation and ECM production

NPDF cells were treated with three different concentrations of TGF-β1 (5, 10, and 20 ng/mL) for 24 hours. The mRNA expression of α-SMA, which is a biomarker representing myofibroblast differentiation, and FBN and type I collagen, biomarkers for ECM, was measured using RT-PCR. The results revealed that the relative mRNA expression of α-SMA, FBN, and type I collagen of NPDF significantly and dose-dependently increased under the stimulation with TGF-β1 (*p* < 0.01 and < 0.001) (Fig. S1).

### Effects of LE for treating TGF-β1-induced NPDF on fibroblast differentiation and ECM production

**Fig. 4:**
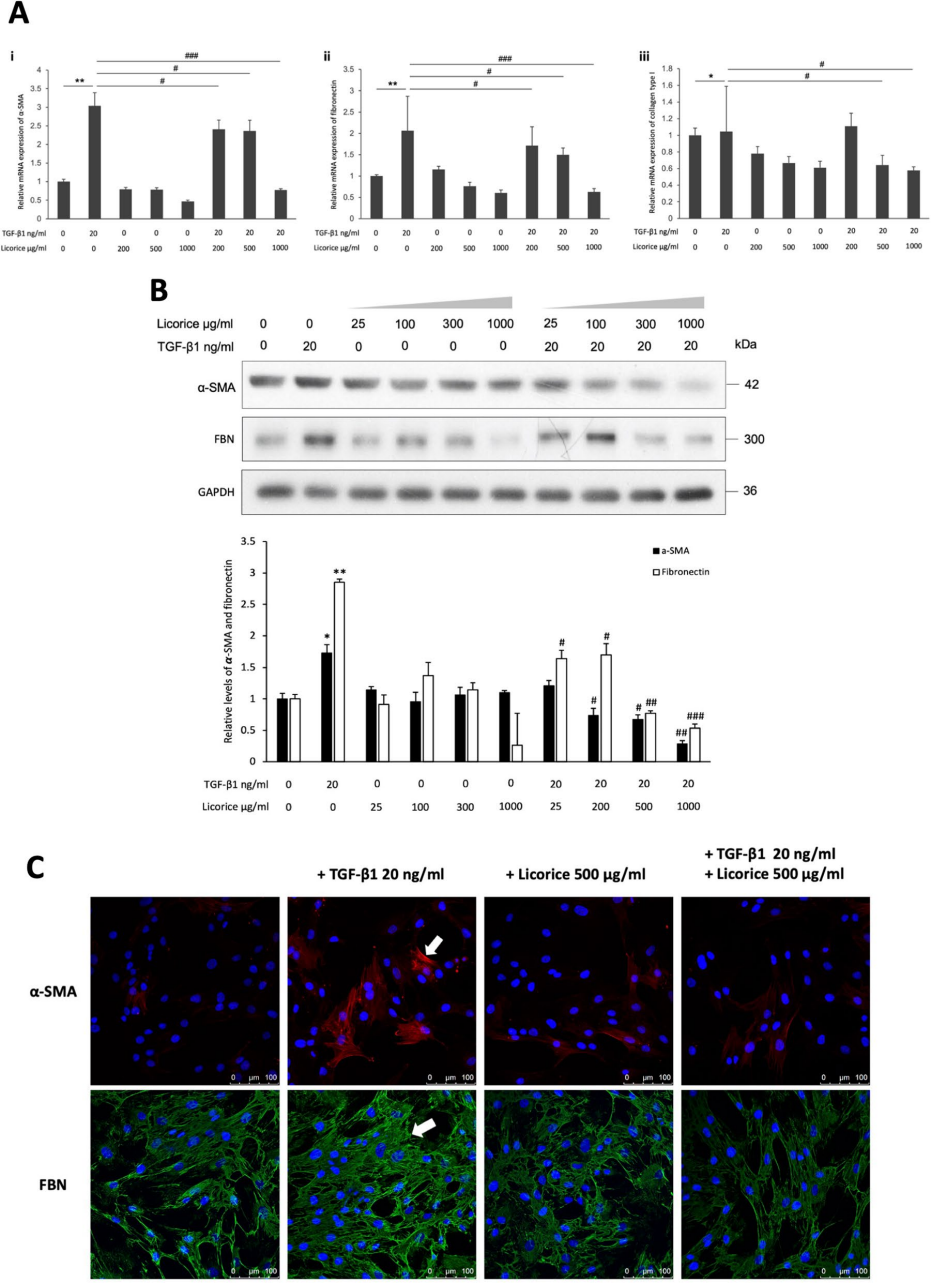
The effects of LE in TGF-β1-stimulated NPDFs for fibroblast differentiation and ECM production. **A** NPDFs with or without TGF-β1 stimulation (20 ng/mL) were treated with LE (200, 500, and 1000 μg/mL) at an increasing concentration, and the mRNA expression levels of α-SMA (i), FBN (ii), and type I collagen (iii) were tested by RT-PCR. **B** LE (25, 100, 300, and 1000 μg/mL) was used to treat NPDFs with or without the activation of TGF-β1 (20 ng/mL). Western blotting was carried out to analyze the protein expressions of α-SMA and FBN, and the results were analyzed by ImageJ software after normalization based on GAPDH. The relative differences between the two groups were statistically analyzed. **C** IF combined with confocal laser microscopy was used to examine the immunofluorescent expression of α-SMA (red fluorescent area indicated by the white arrow) and FBN (green fluorescent area indicated by the white arrow) in NPDFs treated with TGF-β1 (20 ng/mL) alone, LE (500 μg/mL) alone, and the combined TGF-β1 (20 ng/mL) and LE (500 μg/mL)

We examined the therapeutic effect of different concentrations of LE (200, 500, and 1000 μg/mL) under TGF-β1 (20 ng/mL) stimulation for NPDFs using RT-PCR. The results indicated that different concentrations of LE did not increase the mRNA expression of α-SMA, FBN, and type I collagen. However, under the stimulation of TGF-β1, LE could effectively prevent the mRNA expression of α-SMA, which can promote NPDF differentiation and FBN and type 1 collagen for ECM production, and the effect was dose-dependent (Fig. 4A).

Next, to evaluate the effect of LE in treating NPs at the protein level, western blotting was used to analyze the expression of α-SMA and FBN of NPDFs stimulated by TGF-β1 (20 ng/mL) and treated with different concentrations of LE (25, 100, 300 and 1000 μg/mL) for 24 hours. The results revealed that LE alone did not promote NPDF differentiation and increase ECM production. However, under the stimulation of TGF-β1 on NPDFs, LE significantly prevented the protein expression of α-SMA and FBN dose-dependently (*p* < 0.05 and *p* < 0.01) (Fig. 4B).

Furthermore, using IF staining and observation under confocal laser microscopy, it was established that when NPDFs were stimulated by TGF-β1 (20 ng/mL), the production of α-SMA (red fluorescence indicated by white arrow) and FBN (green fluorescence indicated by white arrow) significantly increased. However, under the action of LE (500 μg/mL), it did not increase α-SMA and FBN expressions, but the use of LE (500 μg/mL) to treat TGF-β1-stimulated NPDFs for 24 hours significantly attenuated the expression of α-SMA and FBN (Fig. 4C).

### Functional pathway of LE in treating NPs

**Fig. 5:**
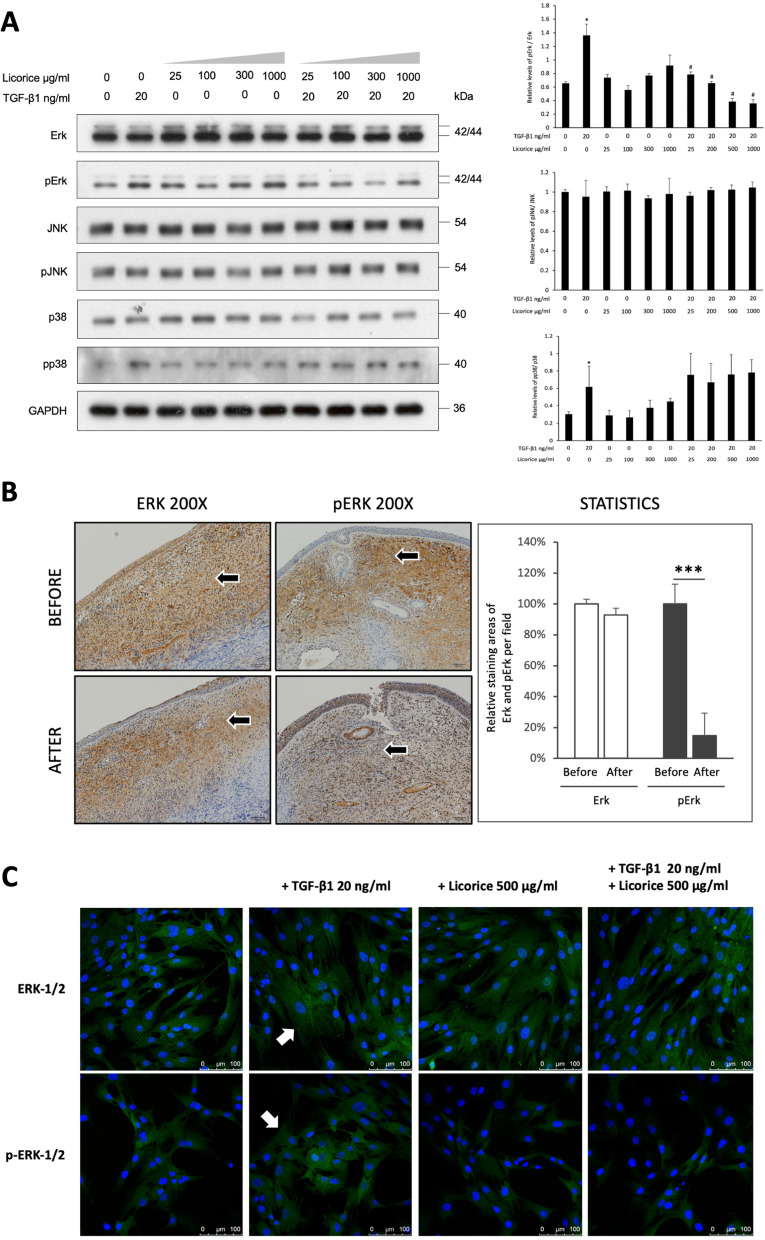
The functional mechanism of LE acting on TGF-β1-stimulated NPDFs. **A** Increasing concentrations of LE (25, 100, 300, and 1000 μg/mL) were used to treat the NPDFs with or without stimulation of TGF-β1 (20 ng/mL) for 24 hours. The expressions of ERK-1/2, p-ERK-1/2, JNK, p-JNK, p38, and p-p38 were measured using western blotting. Western blotting results were analyzed using ImageJ software and corrected with GAPDH. The normalization was performed by dividing the phosphorylated form by the total transcription factors. The relative differences between p-ERK/ERK, p-JNK/JNK, and p-p38/p38 between the two groups were statistically analyzed. **B** The three patients with NPs in this study received polyp biopsies before and 2 weeks after treatment with LE. The staining ERK-1/2 and p-ERK-1/2 were measured using IHC, and the antibody-stained areas were analyzed using the ImageJ software. Statistical analysis was performed (*n* = 3). **C** Under IF staining, confocal laser microscopy was used to examine the immunofluorescent expressions of ERK-1/2 and p-ERK-1 in the NPDFs treated with (1) 20 ng/mL TGF-β1, (2) 500 μg/mL LE, and (3) 20 ng/mL TGF-β1 and 500 μg/mL LE for 24 hours (green fluorescent area indicated by the white arrows)

According to previous NP-associated studies, we selected three key pathways, namely, MAPK/ERK-1/2, JNK, and p38, to explore the functional mechanism by which LE acts in NPDFs [14, 18, 19]. Notably, the NPDF cells were stimulated with TGF-β1 (20 ng/mL) and treated without or with 25, 100, 300 and 1000 μg/mL LE for 24 hours. The reactions were determined using western blotting. With ImageJ software, we did normalization based on the total ERK-1/2, p38 and JNK levels, and got the corresponding statistical analysis after correction. The results revealed that LE significantly inhibited the MAPK/ERK-1/2 pathway, and the effect was dose-dependent (*p* < 0.05) (Fig. 5A).

Next, we confirmed the findings of western blotting with histopathology. The NP specimens of the three patients before and after treatment were stained for ERK and p-ERK. The results showed that the p-ERK levels of NPs significantly decreased after licorice treatment (*p* < 0.001) rather than the ERK levels. Additionally, the pathological tissues’ results were consistent with those of western blotting (Fig. 5B).

Furthermore, in the observation of IF staining and confocal laser microscopy, it was recorded that under the stimulation of TGF-β1 (20 ng/mL), the expression of ERK-1/2 in NPDF was not significantly altered (green fluorescent area indicated by the white arrow), but the expression of p-ERK-1/2 significantly increased (green fluorescent area indicated by the white arrow). LE (500 μg/mL) alone did not affect the expression of the ERK-1/2 and p-ERK-1/2, and the treatment of TGF-β1-stimulated NPDF for 24 hours with LE (500 μg/mL) did not alter the expression of ERK-1/2 but significantly inhibited p-ERK-1/2 (Fig. 5C).

### Effect of LE on cell migration in TGF-β1-stimulated NPDFs

**Fig. 6:**
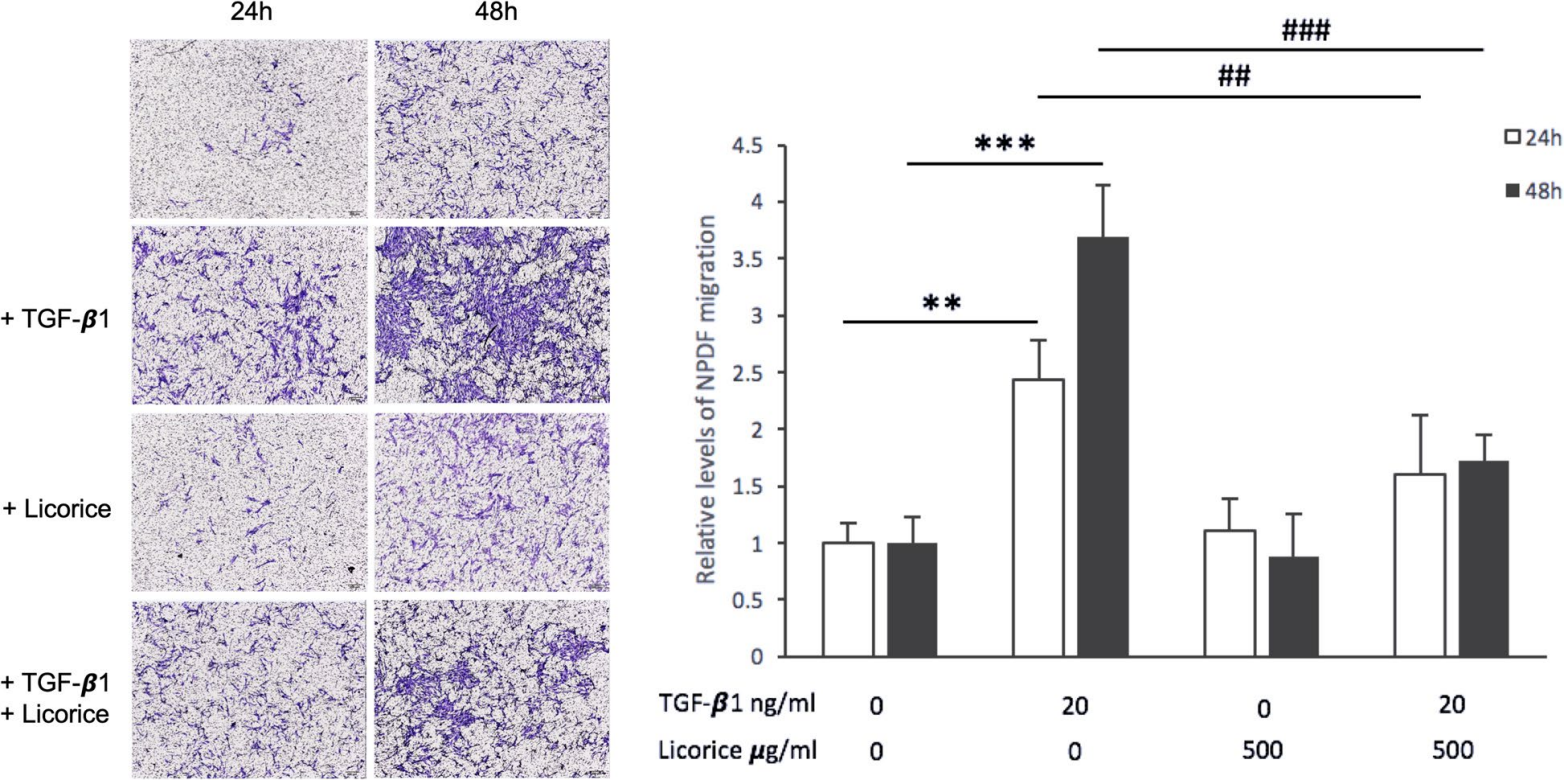
The effect of LE on cell migration in NPDFs. Transwell migration assay was used to analyze the difference between the NPDFs with or without TGF-β1 (20 ng/mL) stimulation and with or without LE (500 μg/mL) treatment for 24 and 48 hours. ImageJ software was used to analyze the stained areas in each result of the transwell migration assay. Additionally, statistical analysis was used to compare the differences between the two groups

The cell migration ability of the fibroblasts is also related to the shape and volume of NPs because the activated fibroblast can generate ECM to increase the interstitial tissue of NPs. Therefore, we analyzed the LE acting on NPDFs in cell migration. In the transwell migration assay, the NPDFs were stimulated by TGF-β1 (20 ng/mL) and a significant increase in cell migration at 24 and 48 hours was observed. However, after adding LE (500 μg/mL) together, cell migration was significantly and effectively suppressed (Fig. 6).

## Discussion

**Fig. 7:**
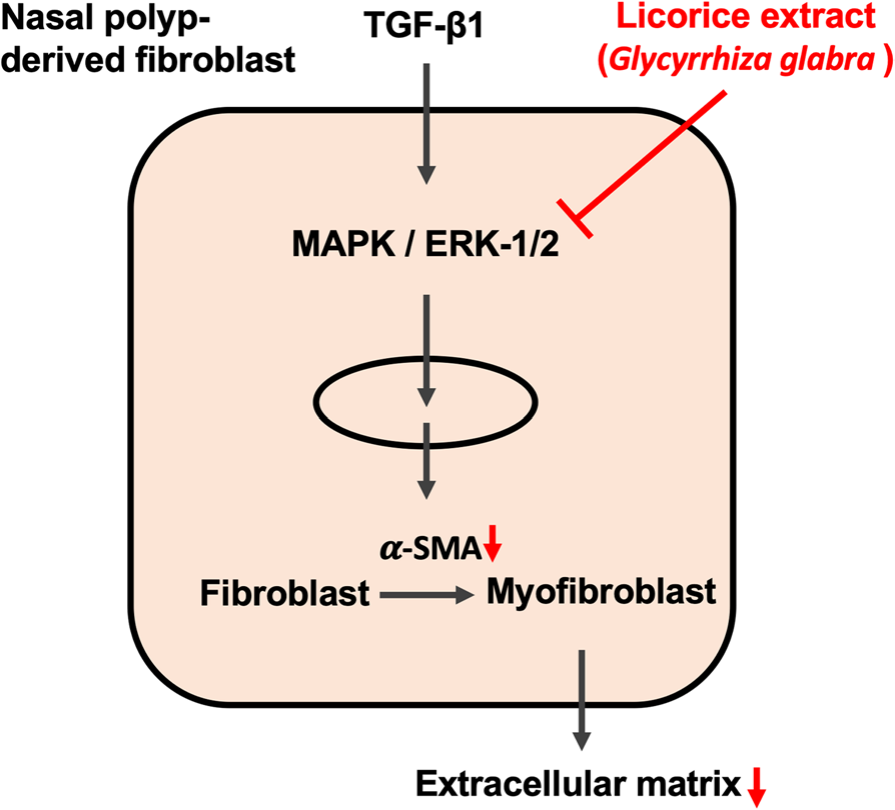
The effect and mechanism of LE (*Glycyrrhiza glabra*) acting on TGF-β1-activated NPDFs. By inhibiting the MAPK/ERK-1/2 signaling pathway, LE prevents downstream α-SMA from differentiating fibroblasts into myofibroblasts and consequently impairs the production of ECM, including FBN and type I collagen

Clinically, we used the LE previously developed to treat rhinitis and sinusitis through nasal irrigation [13] and discovered that this innovative method significantly affects the treatment of NPs. Notably, this study found that after the NPs were treated with LE, the differentiating fibroblast factor (α-SMA) and activation protein (FAP) decreased in the pathological tissue and the collagen (Masson’s trichrome staining) of the polyp tissue also reduced. In addition, the interstitial tissue became relatively sparse. We further used RT-PCR and western blotting to confirm that this LE could inhibit TGF-β1-stimulated NPDF’s α-SMA generation at both mRNA and protein levels to reduce fibroblast differentiation into myofibroblast, which consequently reduced ECM production. A mechanism by which LE treats NPDFs may be achieved by inhibiting the MAPK/ERK pathway, as confirmed by western blotting, IF, and IHC. Additionally, LE inhibited the cell migration of TGF-β1-stimulated NPDFs, which affected the expansion of polyp volume. Therefore, the microscopic findings of this study confirmed the therapeutic results of macroscopic observations in clinical practice (Fig. 7).

Many previous studies have indicated that TGF-β1 is an important stimulator of NP development [16, 17]. Downstream inflammatory signaling pathways such as MAPK/ERK, JNK, and p38 can be initiated after engaging with receptors on the surface of NPDF cells. This signaling further triggers the activation of NF-κB to incorporate into the nucleus and generate α-SMA, which differentiates fibroblasts into myofibroblasts, ultimately leading to the generation of ECM and increasing the macroscopic polyp volume. A few previous studies have pointed out that *Glycyrrhiza glabra* can achieve neuroprotective effects by preventing the inflammatory signaling pathway of ERK-1/2 [21]. Notably, this is similar to our findings using *Glycyrrhiza glabra* to treat the TGF-β1-stimulated NPDFs by inhibiting the ERK-1/2 signaling mediators. Additionally, many studies have documented that the main compound of licorice, glycyrrhizic acid, can attenuate nervous inflammation after spinal cord injury and osteoporosis after menopause by preventing inflammatory pathways such as JNK and p38 [22–24]. In this study, *Glycyrrhiza glabra* had no significant inhibitory effect on p38 and JNK signaling pathways in treating NPDFs. Consequently, these causes might be responsive to some patients with NPs who had relatively poor responses to licorice treatment in the clinic. This phenomenon is worthy of exploration in subsequent studies.

According to related research and treatment guidelines for sinusitis, CRS is categorized into two phenotypes based on whether NPs were observed under endoscopy, namely, CRS with NP (CRSwNP) and CRS without NP. Furthermore, NP is divided into two subtypes based on whether there was obvious eosinophil infiltration of NPs, namely, eosinophilic NP (E-NP) and non-eosinophilic NP (NE-NP) [25, 26]. E-NP is more related to Th2 inflammation. Therefore, a significant increase in cytokines such as IL-4, IL-5, and IL-13 may be observed in polyps. The pathological tissue may reveal significant edema manifestations. Generally, E-NP clinically affects numerous sinuses compared to NE-NP. Notably, NE-NP is more associated with Th1 inflammation. Furthermore, the polyp specimen may have neutrophil infiltration, and the pathological tissue may present as epithelial proliferation and fibrosis changes [25, 26].

The three patients with NPs enrolled in this study showed a high number of eosinophils and inflammatory cell infiltration based on the results of H&E staining. Additionally, it was found that their eosinophils were significantly reduced after 2 weeks of LNI (Fig. 3A). Therefore, we propose that this innovative treatment will significantly affect E-NPs. However, a group of patients with CRSwNP was observed with less clinical effect on this therapy (Fig. 1B). Therefore, follow-up studies should be conducted to analyze the clinical effects of E-NP and NE-NP after receiving licorice treatment. Namely, these results, which include the histopathological findings and differences in NPDFs from E-NP and NE-NP treated with LE, can provide complete understanding of the novel method’s efficacy in CRSwNP treatment.

The fibroblasts used in this study were isolated from the nasal polyp and cultured. In order to investigate whether this LE has the same effect on fibroblasts isolated from normal nasal mucosal tissue. We collected nasal turbinate mucosa from two other patients, and then isolated the fibroblasts in the same way described in this study and performed cell culture. We examined NPDFs and nasal mucosa-derived fibroblasts (NMDFs) under a microscope and found that they differed somewhat in morphology, with the latter being more elongated (Fig. S2). Then we seeded 6 × 10^5^ NMDFs on a 6 cm dish, and then added TGF-β1 (20 ng/mL) or not and treated with or without LE (500 and 1000 μg/mL) for 24 hours according to the groups for Western blotting assay and analyzed by ImageJ software. Fig. S3 showed that TGF-β1 (20 ng/mL) was still effective in activating the fibroblasts isolated from normal nasal mucosa to increase the production of fibronectin, and LE was effective in inhibiting the production of fibronectin in the TGF-β1-stimulated fibroblasts dose-dependently. The findings suggest that LE has the same therapeutic effect on fibroblast isolated from nasal polyp or nasal mucosal tissue.

## Conclusions

Clinical application of nasal irrigation with licorice has a significant effect in treating NPs. According to this study’s histopathology and cell experiments, *Glycyrrhiza glabra* effectively prevents fibroblast differentiation and ECM production in the TGF-β1-stimulated NPDF and attenuates its cell migration. One of the functional mechanisms may be through inhibition of the MAPK/ERK-1/2 signaling pathway to achieve this effect.

## Supplementary Information

**Additional file 1: Fig. S1.** The effect of TGF-β1 on NPDF for fibroblast differentiation and ECM production. The mRNA expressions of α-SMA (i), FBN (ii), and type I collagen (iii) were determined by RT-PCR after the NPDFs were treated with TGF-β1 in increasing concentrations (5, 10, and 20 ng/mL) for 24 hours. **Fig. S2.** Fibroblasts from nasal polyp and nasal mucosa. (A) Fibroblasts isolated from nasal polyp specimens of two patients and cultured, and the cell morphology (black arrow) was observed by optical microscopy. (B) Fibroblasts isolated from nasal turbinate mucosa of two other patients and cultured (black arrow), and the cell morphology appeared to be distinctive from the former. **Fig. S3.** The effect of LE on the expression of extracellular matrix in NMDFs. 6 × 10^5^ nasal mucosa-derived fibroblasts (NMDFs) were seeded on a 6 cm dish and then added TGF-β1 (20 ng/mL) or not and treated with or without LE (500 and 1000 μg/mL) for 24 hours according to the groups for Western blotting assay. The results were analyzed with ImageJ software. TGF-β1 (20 ng/ml) could effectively activate the NMDF to express fibronectin (FBN), and LE was effective in inhibiting the activation dose-dependently.

## Data Availability

If detailed data are required, they can contact the correspondence of the study, Pey-Jium Chang (Email address: a9244@cgmh.org.tw).

## Abbreviations

α-SMA: α-smooth muscle actin
CRS: Chronic rhinosinusitis
CRSwNP: Chronic rhinosinusitis with nasal polyposis
ERK: Extracellular signal-regulated kinase
E-NP: Eosinophilic nasal polyp
JNK: c-Jun N-terminal kinase
LE: Licorice extract
MAPK: Mitogen-activated protein kinase
NP: Nasal polyp
NPDF: Nasal polyp-derived fibroblast
NE-NP: Non-eosinophilic nasal polyp
TGF-β1: Transforming growth factor-beta 1
