# Supplementary material for: Nasal irrigation with licorice extract (Glycyrrhiza glabra) in treating nasal polyps by reducing fibroblast differentiation and extracellular matrix production in TGF-β1-stimulated nasal polyp-derived fibroblasts by inhibiting the MAPK/ERK-1/2 pathway – an in vitro and in clinic study
Source: BMC Complement Med Ther. 2022 Nov 29;22:313. doi: 10.1186/s12906-022-03791-y (PMC9706886; doi:10.1186/s12906-022-03791-y)
Supplement: Supplementary file 1 — Additional file 1: Fig. S1. The effect of TGF-β1 on NPDF for fibroblast differentiation and ECM production. The mRNA expressions of α-SMA (i), FBN (ii), and type I collagen (iii) were determined by RT-PCR after the NPDFs were treated with TGF-β1 in increasing concentrations (5, 10, and 20 ng/mL) for 24 hours. Fig. S2. Fibroblasts from nasal polyp and nasal mucosa. (A) Fibroblasts isolated from nasal polyp specimens of two patients and cultured, and the cell morphology (black arrow) was observed by optical microscopy. (B) Fibroblasts isolated from nasal turbinate mucosa of two other patients and cultured (black arrow), and the cell morphology appeared to be distinctive from the former. Fig. S3. The effect of LE on the expression of extracellular matrix in NMDFs. 6 × 10^5^ nasal mucosa-derived fibroblasts (NMDFs) were seeded on a 6 cm dish and then added TGF-β1 (20 ng/mL) or not and treated with or without LE (500 and 1000 μg/mL) for 24 hours according to the groups for Western blotting assay. The results were analyzed with ImageJ software. TGF-β1 (20 ng/ml) could effectively activate the NMDF to express fibronectin (FBN), and LE was effective in inhibiting the activation dose-dependently. [file 12906_2022_3791_MOESM1_ESM.docx]

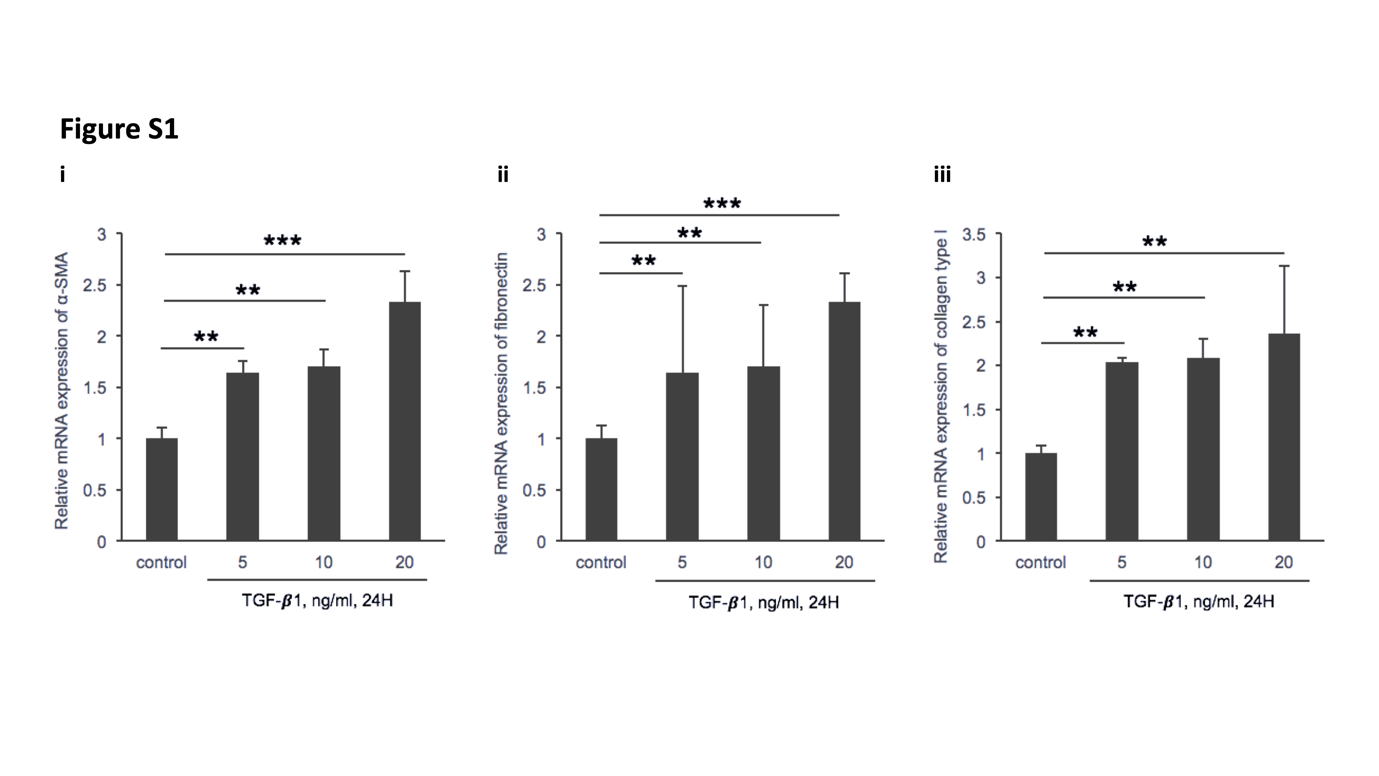


**Figure S1. The effect of TGF-**β**1 on NPDF for fibroblast differentiation and ECM production.**

The mRNA expressions of α-SMA (i), FBN (ii), and type I collagen (iii) were determined by RT-PCR after the NPDFs were treated with TGF-β1 in increasing concentrations (5, 10, and 20 ng/mL) for 24 hours.


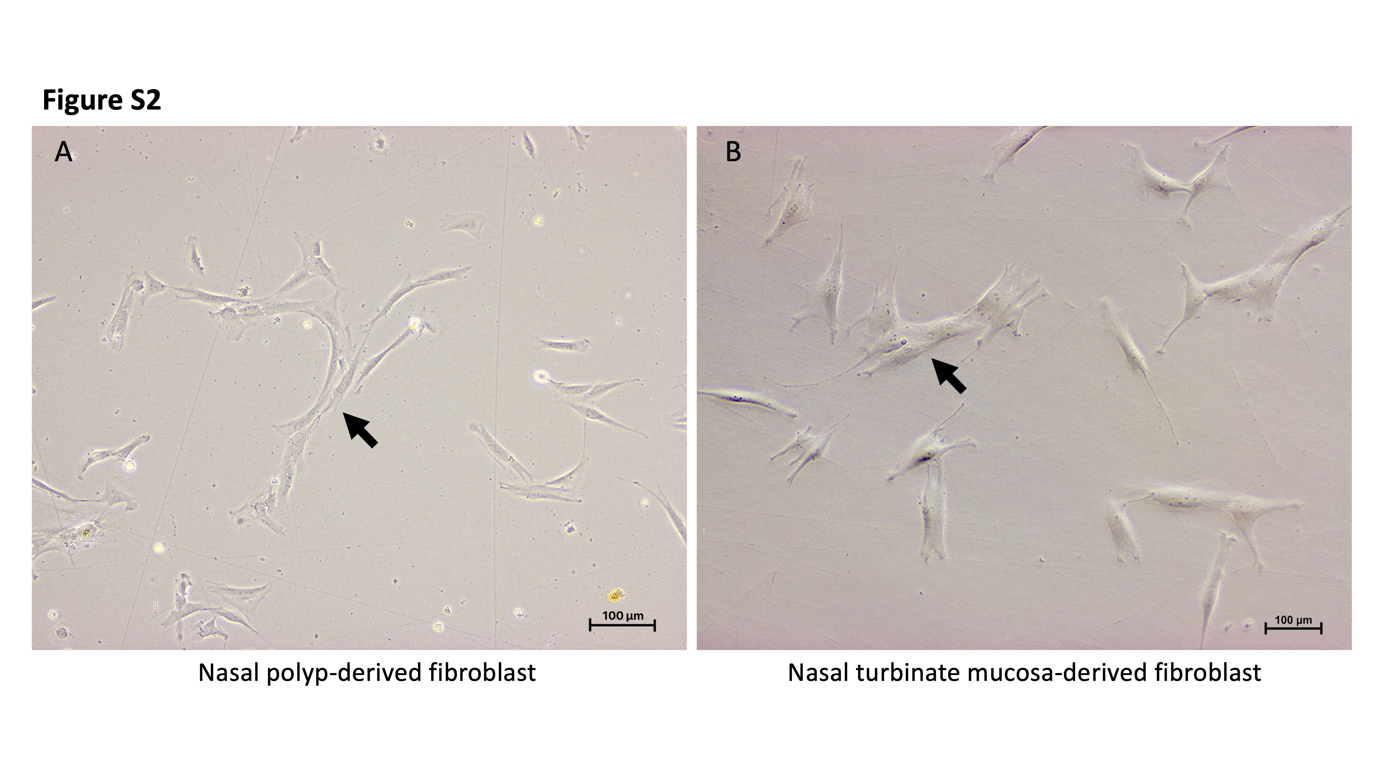


**Figure S2. Fibroblasts from nasal polyp and nasal mucosa.** (A) Fibroblasts isolated from nasal polyp specimens of two patients and cultured, and the cell morphology (black arrow) was observed by optical microscopy. (B) Fibroblasts isolated from nasal turbinate mucosa of two other patients and cultured (black arrow), and the cell morphology appeared to be distinctive from the former.


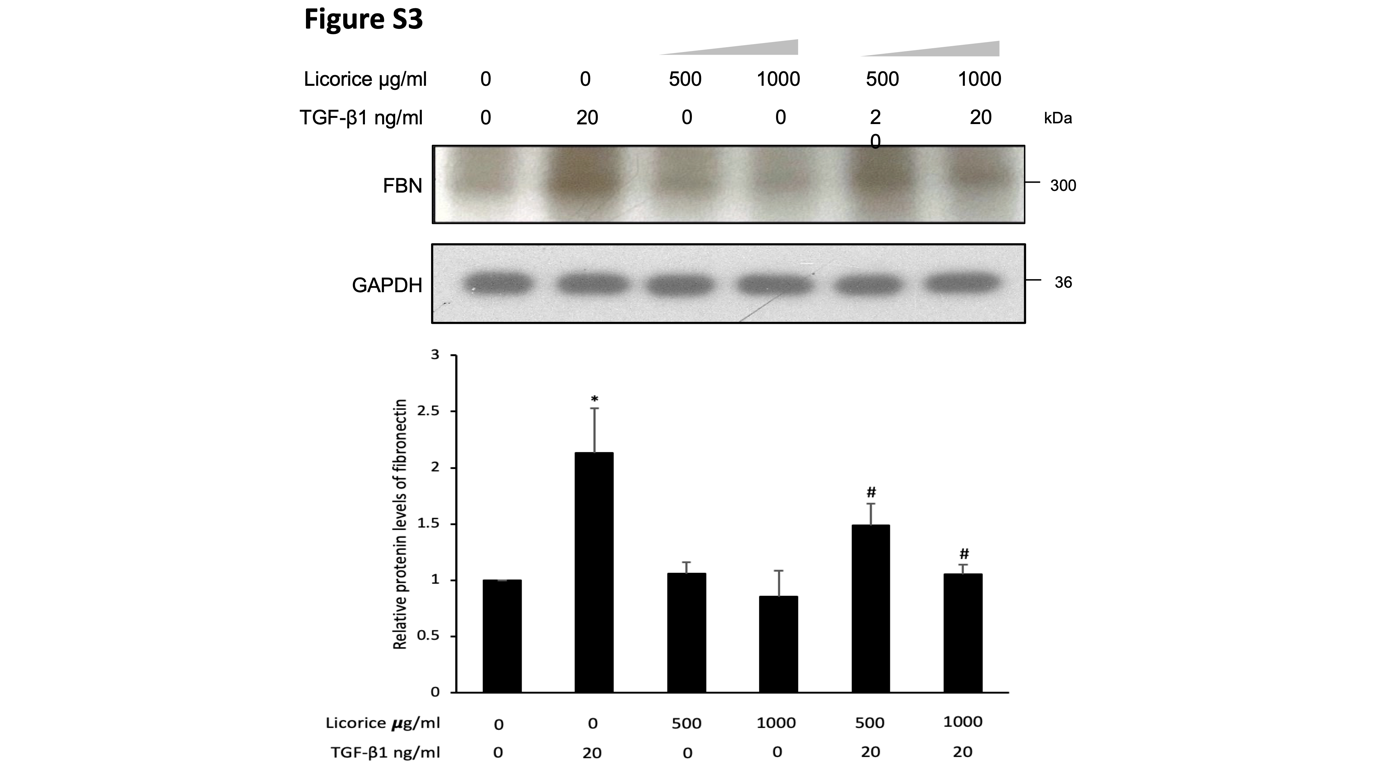


**Figure S3. The effect of LE on the expression of extracellular matrix in NMDFs.** 6 x 10^5^ nasal mucosa-derived fibroblasts (NMDFs) were seeded on a 6 cm dish and then added TGF-β1 (20 ng/mL) or not and treated with or without LE (500 and 1000 μg/mL) for 24 hours according to the groups for Western blotting assay. The results were analyzed with ImageJ software. TGF-β1 (20 ng/ml) could effectively activate the NMDF to express fibronectin (FBN), and LE was effective in inhibiting the activation dose-dependently.
